# Risky movements? Natal dispersal does not decrease survival of a large herbivore

**DOI:** 10.1002/ece3.7227

**Published:** 2021-02-04

**Authors:** Eric S. Long, Duane R. Diefenbach, Clayton L. Lutz, Bret D. Wallingford, Christopher S. Rosenberry

**Affiliations:** ^1^ Department of Biology Seattle Pacific University Seattle WA USA; ^2^ Pennsylvania Cooperative Fish and Wildlife Research Unit Pennsylvania State University U.S. Geological Survey University Park PA USA; ^3^ Southcentral Region Pennsylvania Game Commission Huntingdon PA USA; ^4^ Bureau of Wildlife Management Pennsylvania Game Commission Harrisburg PA USA

**Keywords:** benefit, cost, dispersal, mortality, philopatry, risk, survival, trade‐offs, transfer, white‐tailed deer

## Abstract

Natal dispersal is assumed to be a particularly risky movement behavior as individuals transfer, often long distances, from birth site to site of potential first reproduction. Though, because this behavior persists in populations, it is assumed that dispersal increases the fitness of individuals despite the potential for increased risk of mortality. The extent of dispersal risk, however, has rarely been tested, especially for large mammals. Therefore, we aimed to test the relationship between dispersal and survival for both males and females in a large herbivore. Using a radio‐transmittered sample of 398 juvenile male and 276 juvenile female white‐tailed deer (*Odocoileus virginianus*), we compared survival rates of dispersers and nondispersers. We predicted that dispersing deer would experience greater overall mortality than philopatric deer due to direct transfer‐related risks (e.g., vehicular collision), indirect immigration‐related mortality attributable to colonization of unfamiliar habitat, and increased overwinter mortality associated with energetic costs of movement and unfamiliarity with recently colonized habitat. For both male and female yearlings, survival rates of dispersers (male = 49.9%, female = 64.0%) did not differ from nondispersers (male = 51.6%, female = 70.7%). Only two individuals (both female) were killed by vehicular collision during transfer, and overwinter survival patterns were similar between the two groups. Although dispersal movement likely incurs energetic costs on dispersers, these costs do not necessarily translate to decreased survival. In many species, including white‐tailed deer, dispersal is likely condition‐dependent, such that larger and healthier individuals are more likely to disperse; therefore, costs associated with dispersal are more likely to be borne successfully by those individuals that do disperse. Whether low‐risk dispersal of large mammals is the rule or the exception will require additional research. Further, future research is needed to evaluate nonsurvival fitness‐related costs and benefits of dispersal (e.g., increased reproductive opportunities for dispersers).

## Graphical abstract

Dispersal is widely considered a particularly risky movement behavior, exposing dispersers to dangers like roadkill and predation. From a sample of 675 radio‐collared, juvenile white‐tailed deer, we found that dispersers and nondispersers survived similarly, with only two deer dying during dispersal. Thus, white‐tailed deer seem to mitigate successfully risks associated with long‐distance movements.

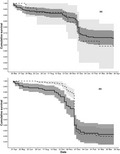

## 1 INTRODUCTION

Animal movements represent a cost–benefit trade‐off, such that fitness may be increased (e.g., competition avoidance, resource acquisition) or decreased (e.g., energy expenditure, exposure to predators) by movement behavior (Fahrig, 2007; Lima & Dill, 1990; Ronce, 2007). Natal dispersal, or movement of individuals away from birth site to location of first potential reproduction (Howard, 1960), is a critical life‐history strategy affecting many ecological and evolutionary processes (Bullock et al., 2002; Clobert et al., 2012). Dispersal, however, is typically considered a particularly risky behavior, especially in human‐modified landscapes (Shaw et al., 2014; Solomon, 2003; Zollner & Lima, 2005).

Fitness consequences of dispersal remain relatively poorly understood in many systems. Despite substantial differences in dispersal patterns (e.g., active vs. passive dispersal), conceptual similarities exist such that three phases of natal dispersal are often recognized: emigration (i.e., departure from natal range), transfer (i.e., movement from natal range to a distinct, nonoverlapping adult range), and immigration (i.e., establishment of adult range; Andreassen et al., 2002; Jongejans et al., 2015). Although successful long‐distance dispersers often demonstrate increased fitness relative to philopatric conspecifics (Bowler & Benton, 2005; Larsen & Boutin, 1994; Stephens et al., 2005), dispersers may incur substantial costs in each dispersal phase (Bonte et al., 2012), for instance, fitness benefits of dispersal trade‐off with energetic costs of predeparture exploratory movements (Debeffe et al., 2013), ambulatory costs during transfer (Benoit et al., 2020), opportunity costs associated with lost familiarity of natal habitat (Part, 1991), and direct mortality risks of predation (Bonnet et al., 1999) or vehicular collision (Real & Mañosa, 2001). Further, estimating the balance of cause‐specific costs and benefits can be complicated by the observation that dispersal within a population may have multiple ultimate causes (Long et al., 2008; Stenseth & Lidicker, 1992), and highly modified habitats may yield suboptimal movement patterns (Fahrig, 2007; Shaw et al., 2014).

For actively dispersing vertebrates, costs tend to be most pronounced in the transfer and immigration phases of dispersal (Bonte et al., 2012). Specifically, long‐distance dispersal increases direct mortality risks during transfer and increases energy expenditure, which may decrease postdispersal survival. For instance, Massemin et al. (1998) demonstrated increased mortality of barn owls (*Tyto alba*) in France due to vehicular collision during dispersal, and Bonnet et al. (1999) similarly attributed young‐of‐the‐year snake mortality in France to dispersal movements. Further, using animal‐borne biologgers in France, Benoit et al. (2020) demonstrated significantly greater energy expenditure in dispersing roe deer (*Capreolus capreolus*) relative to philopatric conspecifics, and Johnson et al. (2009) attributed increased mortality of dispersing martens (*Martes americana*) in Ontario, Canada, to increased energy expenditure.

White‐tailed deer (*Odocoileus virginianus*) are large mammals whose dispersal patterns have been relatively well‐studied and may serve as a model for large‐mammal dispersal. For both sexes, dispersal primarily occurs in yearlings (i.e., 1‐year‐olds), and like most mammals, dispersal is male‐biased, with yearling male emigration rates of approximately 50%–80% (Long et al., 2005). Dispersal in females is less common, with yearling female dispersal rates of only 3%–50% (Lutz et al., 2015). Greenwood (1980) attributed sex‐biased dispersal of vertebrates to sex‐specific ultimate causes, as we have suggested for white‐tailed deer (Long et al., 2008; Lutz et al., 2015). Thus, fitness consequences of potentially risky dispersal likely affect sexes differently.

Although causes of dispersal are becoming clearer, costs associated with dispersal of large mammals remain poorly understood (Bonte et al., 2012). Generally, costs are assumed to be high due to energetic expenditure and direct threat of transfer‐related mortality (e.g., vehicular collision, exposure to predators); however, few studies of large mammals have documented dispersal‐related risks. In white‐tailed deer, dispersal has been predicted to increase mortality (McCoy et al., 2005; Nelson & Mech, 1986; Roseberry & Klimstra, 1974), but this prediction has rarely been tested. To our knowledge, only two prior studies of large mammals (both white‐tailed deer) have compared mortality of dispersers and nondispersers: Nixon et al. (2001), working in highly fragmented agricultural region of Illinois (USA), suggested that dispersal increased mortality risk, but Haus et al. (2019), working in Delaware (USA), suggested that dispersal status was not a significant predictor of mortality for yearling male white‐tailed deer.

Here, we present dispersal‐related mortality for both sexes of white‐tailed deer through all phases of dispersal. To test for mortality risks associated with dispersal, we compared time‐specific survival of dispersing and philopatric yearling male and female white‐tailed deer from multiple populations. We predicted that dispersing deer would experience greater overall mortality than philopatric deer due to (a) direct transfer‐related risks such as vehicular collision, (b) indirect immigration‐related mortality attributable to recent colonization of unfamiliar habitat, and (c) increased winter mortality associated with energetic costs of movement and unfamiliarity with recently colonized habitat. Female white‐tailed deer transfer movements are longer and more tortuous than male movements (Lutz et al., 2016; Nixon et al., 2001); therefore, we predicted direct transfer‐related risks would be greater in female dispersers. Further, yearling female dispersal is generally completed by early summer (Lutz et al., 2015), whereas many yearling males disperse in fall just prior to or concurrent with hunting seasons (Long et al., 2008; Peterson et al., 2017); therefore, we predicted that male dispersers would bear greater indirect risks associated with recent colonization.

## 2 METHODS

### 2.1 Study areas

**FIGURE 1:**
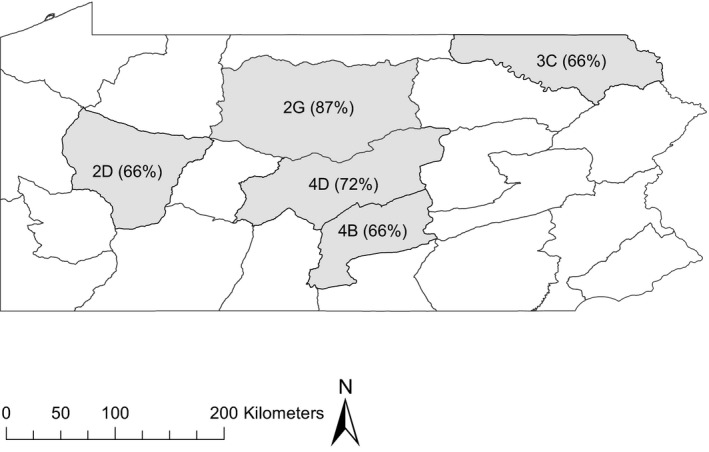
Map of Wildlife Management Units (WMU) in the state of Pennsylvania (USA), highlighting the five WMUs in which deer were captured for this study. Values in parentheses indicate percent forest cover for each WMU

We estimated dispersal parameters of deer in five Wildlife Management Units (WMUs) in Pennsylvania, USA (Figure 1). These WMUs represented diverse physiographic regions, including the Pittsburgh Low Plateau of western Pennsylvania (WMU 2D), the Deep Valleys section of the Appalachian Plateau in northcentral Pennsylvania (WMU 2G), the Glaciated Low Plateau section of the Appalachian Plateau in northeastern Pennsylvania (WMU 3C), and the Ridge and Valley Province of central Pennsylvania (WMUs 4B and 4D). All study areas experience a temperate climate; elevations do not exceed 820 m; and deer within these areas do not migrate to winter ranges (Long et al., 2005; Lutz et al., 2015). Hunting of both male and female deer is common in all areas, with limited hunting beginning the first week of October and most hunting beginning the third week of November and ending by the third week of January (Wallingford et al., 2017). To our knowledge, natal and adult ranges of all deer included in this study comprised areas open to hunting. Further, across the study areas, fawns are commonly depredated by coyotes (*Canis latrans*), black bears (*Ursus americanus*), and bobcats (*Lynx rufus*; Vreeland et al., 2004); however, predation of yearling and older deer is uncommon (Wallingford et al., 2017). Winter mortality is relatively uncommon, typically occurring Feb–Mar, and annual survival of yearling and adult deer outside hunting is >90% (Wallingford et al., 2017).

### 2.2 Deer capture and monitoring

From 2002 to 2004, we captured and radio‐marked 398 juvenile males in WMUs 2D and 4D, with capture conducted in each WMU each year. Of these, 381 were equipped with very high frequency (VHF) radio‐transmitters and were located at least weekly (average number of yearling locations ± SE = 50.4 ± 4.5); 17 were equipped with global positioning system (GPS) radio‐transmitters that recorded positions at least twice daily (914.6 ± 88.2). From 2005 to 2010, we captured and radio‐marked 276 juvenile females in WMUs 2D, 2G, 3C, and 4B, again with capture conducted in multiple WMUs each year. Of these, 245 were equipped with VHF transmitters and located at least weekly (64.0 ± 3.8); 32 were equipped with GPS transmitters that recorded position at least daily (1,139.2 ± 304.5). Capture occurred during winter through early spring, ending by 23 Apr for males and 26 Mar for females. At time of capture, juvenile deer were approximately 7–10 months old. For both male and female white‐tailed deer, natal dispersal prior to 11 months of age is rare; thus, capture between December and April decreased the likelihood of capturing juveniles that had already dispersed (Marchinton & Hirth, 1984; Vreeland et al., 2004). Deer were captured January–April using Clover traps, drop‐nets, rocket‐nets, and net‐gun from helicopter. Additional capture, monitoring, and study area details are provided in Long (2005) and Lutz (2015).

### 2.3 Analytical methods

To test for survival differences between dispersers and nondispersers, we estimated survival for males and females using known‐fate models in Program MARK v. 6.2 (White & Burnham, 1999). We modeled males and females in separate analyses because data collection for the sexes did not overlap in time or, with the exception of WMU 2D, in space. Due to estimability and sample size issues, we pooled study areas and years within sex. Thus, for each sex, we developed two candidate models of annual survival: a null model that combined dispersers and nondispersers and a group model that estimated weekly survival separately for dispersers and nondispersers. Individuals were entered into the analysis after the completion of all capture efforts, and weekly survival was estimated through 22 Apr of the following year. Because capture of females ended 1 month earlier than capture of males, the null postcapture model generated 52 and 56 weekly survival estimates for males and females, respectively (Table 1). Additionally, to investigate potential relationship of dispersal distance on mortality, dispersal distance was included as an individual covariate in all models. Dispersal distance was calculated as straight‐line distance between median *x* and *y* predispersal and postdispersal coordinates (Haus et al., 2019; Kenward et al., 2002; Peterson et al., 2017). We used Akaike's information criterion corrected for sample size (AIC_c_) to select the most parsimonious model of survival (Burnham & Anderson, 1998).

**TABLE 1:** Model selection results for weekly survival rates of yearling (1–2 years old) white‐tailed deer in Pennsylvania, USA

Sex	Period	Begin date	*N* ^a^	Model	*K* ^b^	Log(l)^c^	AIC_c_ ^d^	ΔAIC^e^	Akaike weight
Male	Postcapture	23 Apr	398	Null	52	1,120.3	1,224.7	0.0	1.00
				Group	104	1,062.0	1,271.7	46.9	0.00
	Postdispersal	19 Nov	253	Null	22	543.5	587.8	0.0	1.00
				Group	44	529.2	617.7	29.9	0.00
	Hunting	1 Oct	306	Null	15	827.1	857.3	0.0	0.87
				Group	30	800.5	861.1	3.8	0.13
Female	Postcapture	26 Mar	276	Null	56	708.3	820.8	0.0	1.00
				Group	112	683.1	909.3	88.3	0.00
	Postdispersal	2 Jul	225	Null	41	499.7	582.1	0.0	1.00
				Group	82	484.3	650.0	67.9	0.00
	Hunting	1 Oct	220	Null	15	424.8	455.0	0.0	1.00
				Group	30	415.1	475.8	20.8	0.00

Note

Models estimated survival from the begin date through 22 Apr of the following year. Survival of dispersers and nondispersers was modeled together in null models and separately in group models.

^a^ Number of yearling deer entered into each model.

^b^ Number of parameters.

^c^ −2 × Log likelihood.

^d^ Akaike's information criterion adjusted for sample size.

^e^ Difference in AIC_c_ for the current model relative to the model with the lowest AIC_c_.

Because dispersal movements were not observed before mid‐April, deer were censored from analysis if they died before this period or if we were unable to determine their fate. We investigated all mortalities to determine cause of death, which included vehicular collision, starvation, disease, predation, legally killed, and illegally killed (Wallingford et al., 2017). If we could not immediately determine cause of death, we submitted the carcass for necropsy to the Pennsylvania State University Animal Diagnostic Laboratory (University Park, Pennsylvania).

Dispersers were identified as yearling deer that demonstrated permanent emigration from natal range to a distinct adult range that did not overlap the natal range, based on 95% minimum convex polygon range estimates for natal and adult ranges (Haus et al., 2019; Kenward et al., 2002; Peterson et al., 2017). Date of dispersal initiation was defined as the first date that a deer was located outside its natal range without returning or, alternatively, the first date we failed to locate a deer within its natal range and subsequently located it within a distinct, nonoverlapping adult range.

In addition to testing an annual (i.e., postcapture) model of survival, we also performed two subsequent analyses over shorter timescales. In the first, to investigate potential mortality differences independent of actual transfer movements, we began survival analyses after most dispersal‐related movements had ceased. For yearlings, 95% of natal dispersal was completed by 19 Nov for males and 2 Jul for females (Long et al., 2008; Lutz et al., 2015). Therefore, to control for any transfer‐related mortality and to compare postsettlement survival of dispersers and nondispersers, we used these two dates as starting dates for all males and females, respectively, and continued weekly survival analyses through 22 Apr of the year following capture. Thus, for postdispersal analyses, null models generated 22 and 41 weekly survival estimates for males and females, respectively (Table 1). In this way, by beginning survival comparison after transfer was completed, postdispersal analyses could more directly test our second two predictions (i.e., indirect mortality related to unfamiliarity with recently colonized habitat and increased winter mortality related to energetic costs of long‐distance movement.)

Finally, hunting‐related mortality was great for both males and females, and hunting is the greatest source of mortality for white‐tailed deer in Pennsylvania (Norton et al., 2013). Dispersers could have demonstrated greater hunting‐related mortality due to relative unfamiliarity with new home ranges. Therefore, to focus on potential differences in hunting‐related mortality of dispersers and nondispersers, in the third analysis we began estimating weekly survival on 1 Oct for all deer, which corresponded to the beginning of hunting. Similar to previous models, we wanted to include postemigration costs through the potentially risky, resource‐poor winter period; therefore, weekly survival was modeled through 22 Apr of the year following capture. Thus, because start and end date were identical for both sexes in the hunting model, null models generated 15 weekly survival estimates for both males and females (Table 1).

## 3 RESULTS

**FIGURE 2:**
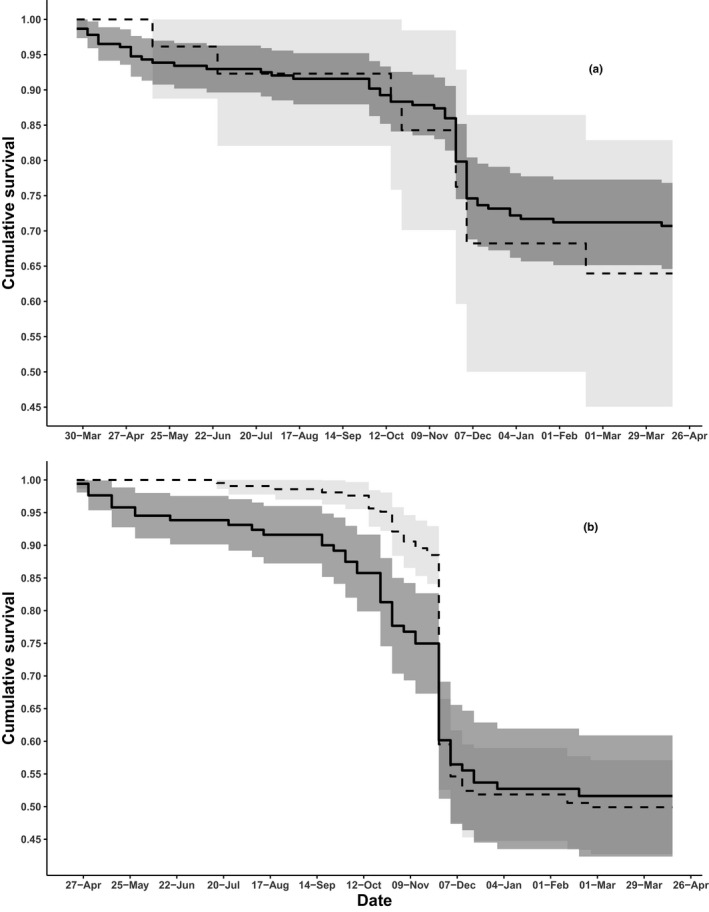
Weekly known‐fate survival estimates (±95% CIs) for 276 yearling (1–2 years old) female (a) and 398 yearling male (b) white‐tailed deer in Pennsylvania, USA. Models begin postcapture (26 Mar for females, 23 Apr for males) and continue through 22 Apr of the following year. For both females and males, no difference was detected in survival between dispersing (black lines) and nondispersing (gray lines) deer

**FIGURE 3:**
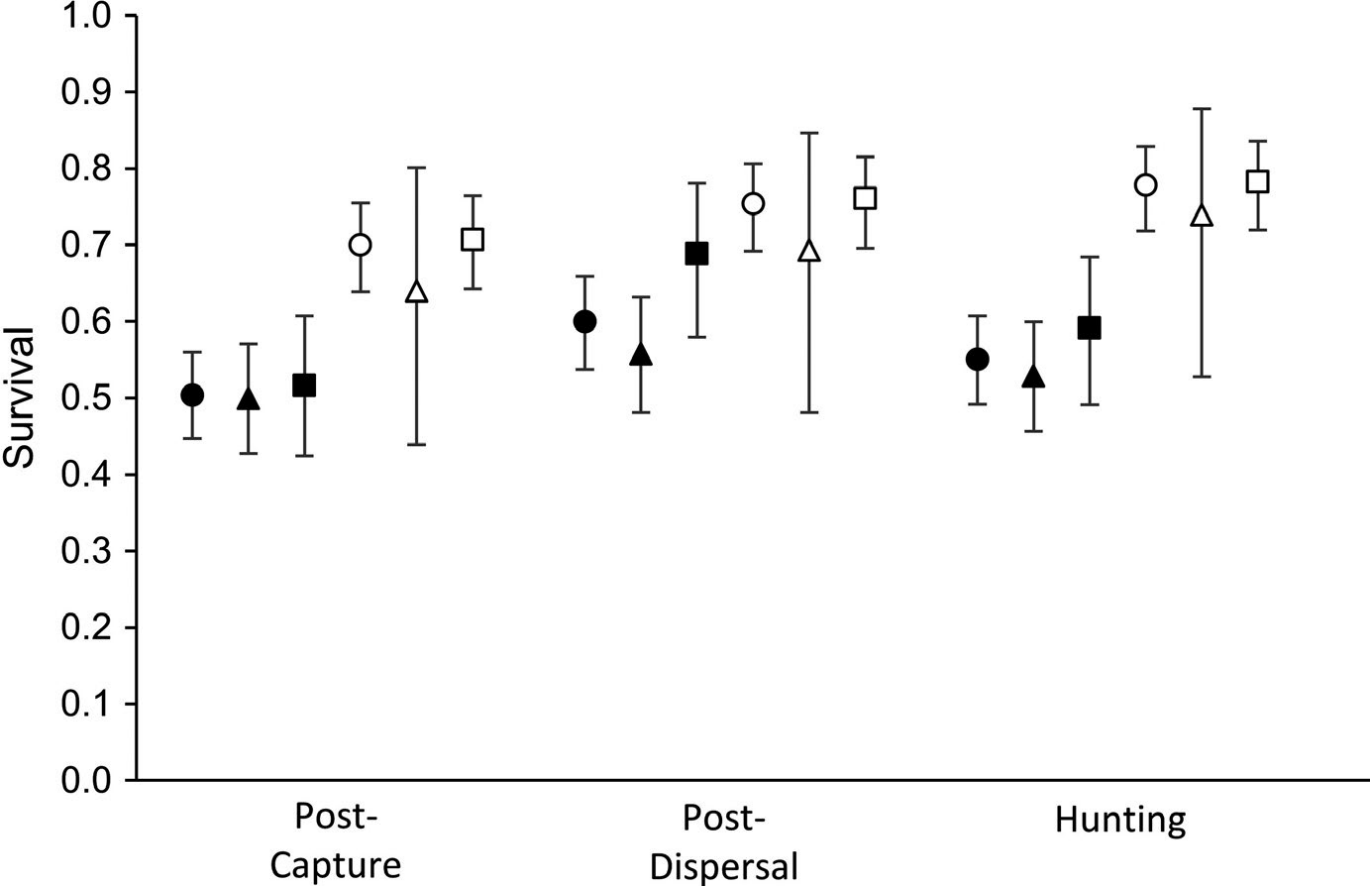
Cumulative weekly survival estimates (±95% CI) for yearling (1–2 years old) male (solid) and female (open) white‐tailed deer in Pennsylvania, USA. To investigate potential temporal differences in survival by dispersal status, three periods were modeled: following winter capture, following 95% of yearling dispersals, and hunting season (beginning 1 Oct). All models estimated cumulative survival through 22 Apr of the following year. In each case, the null model (circles) was more parsimonious than models that estimated survival of dispersers (triangles) and nondispersers (squares) separately

Of the 398 yearling male deer included in the model, 226 dispersed. Of 152 male mortalities, 95 were dispersers and 57 were nondispersers. Of the 276 yearling females included, 27 dispersed, and 86 female mortalities comprised nine dispersers and 77 nondispersers. For both male and female yearling deer, annual survival functions did not differ between dispersers and nondispersers (Figure 2), as the most parsimonious model did not include the grouping factor of dispersal status (AIC_c_ weight = 100%; Table 1). For males, cumulative weekly survival from end of capture through 22 Apr of the following year was 0.503 (95% CI: 0.447–0.560), and when estimated separately, cumulative annual survival differed only 0.017 between dispersers and nondispersers (Figure 3). For females, cumulative weekly survival was 0.700 (95% CI: 0.639–0.755; Figure 3). No evidence for difference between survival of dispersing and nondispersing females was detected, but the point estimate of disperser survival was 0.067 lower than nondisperser survival (Figure 2).

Point estimates for betas of the distance covariate suggested that greater dispersal distances decreased survival, as this parameter covaried negatively with survival for both males (−0.0021, SE = 0.0133) and females (−0.0079, SE = 0.0168). However, 95% CI estimates of this parameter overlapped zero for both males (−0.0281 to 0.0240) and females (−0.0409 to 0.0250), suggesting that any negative effect of dispersal distance on survival was relatively weak.

In highly modified landscapes such as our study areas, vehicular collision provides a potential mortality risk for deer traveling long distances. However, despite observing 253 dispersal movements, we observed only two vehicle‐related mortalities during transfer movements (both female; one automobile collision, one train collision). Considering dispersers and nondispersers together, we attributed 31 yearling male mortalities and 19 additional yearling female mortalities to automobile collision, but none of these mortalities occurred during dispersal.

Further, when analyzed over shorter periods that did not include transfer movements, survival of dispersers similarly showed no difference from survival of nondispersers (Figure 3). The most parsimonious model in each case did not include a grouping effect of dispersal status, and in each case, group models that estimated survival of dispersers and nondispersers separately received very little weight (Table 1). Thus, regardless of whether analyses included dispersal movements, began after establishment of adult range, or isolated the period of peak mortality (i.e., fall hunting), no differences in survival were detected between dispersing and nondispersing yearling deer.

## 4 DISCUSSION

Despite a large sample of male and female white‐tailed deer, we failed to find evidence of direct, survival‐related risks associated with dispersal for either sex, as dispersers and nondispersers survived at similar rates. This finding does not necessarily indicate cost‐free or even low‐cost dispersal, as fitness‐related costs may extend beyond measures of survival. For instance, even after successful immigration, dispersers could potentially experience reduced reproductive opportunities (Cant et al., 2001; Part, 1991). However, contrary to predictions, dispersal in white‐tailed deer does appear to be a relatively low‐risk behavior.

As in many other vertebrates, dispersal in white‐tailed deer is strongly sex‐biased, and we observed greater dispersal of males than females. But if dispersal is not particularly risky for females, why did so few disperse? Although the survival consequences of dispersal are similar, the ultimate causes of dispersal in white‐tailed deer differ greatly between the sexes and seem to relate to reproductive, rather than survival, components of fitness. Male white‐tailed deer disperse to reduce the probability of inbreeding when abundance of adult females is great and to reduce intrasexual competition when abundance of adult males is great (Long et al., 2008; Shaw et al., 2006). Thus, especially if male dispersal is a low‐risk behavior, benefits of dispersal likely frequently outweigh costs, which would ultimately select for dispersal. Consistent with this, male dispersal movements in our study were quick (often <12 hr), direct, and relatively short (median dispersal distance in our system = 5.9 km; Long, 2005; Long et al., 2010), although dispersal movements in more agricultural landscapes are often longer in duration and distance (Anderson et al., 2015; Springer, 2017). These findings suggest that males have adapted efficient dispersal strategies, and like our study, Haus et al. (2019) found no effect of dispersal on yearling male white‐tailed deer survival in Delaware, USA.

Despite being relatively low risk, female dispersal movements of white‐tailed deer remained relatively rare. As Greenwood (1980) suggested, inbreeding is minimized if one sex disperses and the other remains philopatric; thus, inbreeding avoidance likely does not ultimately cause female deer dispersal and may select for philopatry through increased reproductive success. Dispersal that has been observed in females has been attributed to density‐dependent limitation of parturition sites (Lutz et al., 2015); thus, female dispersal is likely a conditional strategy that is more rarely adopted than male dispersal. Female white‐tailed deer dispersal movements seem to be longer and more tortuous than male movements (Lutz et al., 2016; Nelson & Mech, 1992; Nixon et al., 2001). For example, median yearling female straight‐line dispersal distance in our system (15.3 km) was more than 2.5 times greater than median male dispersal distances (Lutz, 2015), and actual distance traveled across the landscape during transfer was certainly longer, especially for females. Although dispersal distance was not a strongly predictive covariate for survival in either sex, the ratio of female to male beta estimates for dispersal as a covariate was 3.84:1, suggesting that increasing dispersal distance may increase risk for females more than males. Determining whether the circuitous, longer‐distance dispersal pattern typical of female white‐tailed deer represents an inefficient search strategy resulting from low selection pressure for dispersal, or a fundamentally different transfer strategy than males’ direct paths, will require additional research. Further, with GPS transmitters capable of more frequent fixes, future studies could incorporate path analysis of dispersers to investigate whether path length and tortuosity of transfer movements influence survival.

In addition to dispersal‐related transfer movements, male and female white‐tailed deer have also been shown to demonstrate temporary, round‐trip excursive forays, which could also potentially increase mortality risk (Jacobsen, 2017; Lutz et al., 2016). These forays are often of short duration but may last 2 weeks or more (Springer, 2017). As such, some mortalities that occur during forays outside natal home ranges may be incorrectly attributed to dispersal‐related mortality, and if animals die during movement without establishing a distinct adult range, distinguishing between forays and dispersal‐related transfers becomes impossible. However, as we have shown, transfer‐related mortality prior to establishing an adult range was low in our system. Further, of deer that did establish adult ranges distinct from natal ranges, >92% of males and >96% of females were recorded in adult ranges for >14 days prior to mortality, suggesting little potential for the inadvertent inclusion of excursive deer in our models of dispersal‐related survival.

Further, although we detected no effect of dispersal on survival for females, due to the rarity of female dispersal we were able to observe only 27 dispersal events. The only transfer‐related vehicular‐collision mortalities were observed in females, and annual point estimates for survival were 6.7% lower for dispersers than nondispersers. Because females, on average, disperse longer distances than males in the same landscape, female dispersal may incur relatively greater costs and expose females to greater risk, but direct risks associated with dispersal seemed to be rare in both sexes. Further, although road‐crossings do frequently occur during dispersal, both males and females seem actively to reduce risk by terminating movements on the near side of major roadways and other potentially dangerous barriers (Long et al., 2010; Lutz et al., 2016).

In addition to direct risks associated with transfer movements, we continued survival analyses through the following April to examine potential delayed costs and indirect risk of dispersal. In addition to hunting, winter mortality of white‐tailed deer can be high (DelGiudice et al., 2002), and energetically costly movements prior to winter could increase winter mortality. For instance, male deer are known to increase their movement rates in late fall and early winter as they search for mates (Long et al., 2013; Whitman et al., 2018), and these activities can increase mortality after the breeding season (Ditchkoff et al., 2001). Benoit et al. (2020) showed that transfer‐related movements of roe deer in France resulted in 22% more energy expenditure for dispersers relative to nondispersers, but any increased energetic costs of dispersal did not apparently translate to decreased survival in our study. Similarly, Whitman et al. (2018) did not see increased white‐tailed deer mortality with increased breeding‐related movements in New York, USA.

However, white‐tailed deer are habitat generalists, and because dispersal patterns differ across landscapes (Long et al., 2005; Lutz et al., 2015; Nixon et al., 2007), dispersal risk may vary as well. In a highly modified and fragmented agricultural landscape with sparse, patchily distributed forest covering 1.6%–20% of the landscape, Nixon et al. (2001) found reduced survival in dispersing white‐tailed deer in Illinois, USA. Nixon et al. (1991, 2001) observed greater female dispersal rates, lower male dispersal rates, and greater dispersal distances than other studies of white‐tailed deer from less fragmented forested habitats. Decreased survival of dispersers was attributed to unfamiliarity with newly settled habitat, described as heavily hunted “dispersal sinks,” wherein survival beyond winter was unlikely (Nixon et al., 1991, 2001). If adult habitat comprised less cover than the forested patches where capture was concentrated, movement to subprime habitat likely increased mortality because forest patches serve as important refuges from hunting in agricultural landscapes (Foster et al., 1997). Further, much of the natal area was located inside reserves with no hunting, and increased mortality was associated with any movements outside refuges (Nixon et al., 1991, 2001). Similarly, Rosenberry et al. (1999) found that emigration from an area with restricted hunting increased yearling male white‐tailed deer mortality in Maryland, USA. Therefore, dispersal from refuges likely increases risk.

In other mammals, such as red fox (*Vulpes vulpes*) in England, banner‐tailed kangaroo rats (*Dipodomys spactabilis*) in Arizona, USA, and American martens in Ontario, Canada, increased dispersal distance has been associated with increased mortality (Harris & Trewhella, 1988; Johnson et al., 2009; Jones, 1988). Although we did not detect a significant effect of dispersal distance on survival, Haus et al. (2019) found decreased mortality with increasing dispersal distance in yearling male white‐tailed deer. As in our study system, hunting was the largest source of mortality, and vehicular collision was relatively minor, although they did not specify if any of the observed vehicular collisions occurred during transfer. Like other studies of white‐tailed deer (McCoy et al., 2005; Shaw et al., 2006), Haus et al. (2019) showed that dispersing males were larger than philopatric males, suggesting condition‐dependent dispersal. In this way, it is possible that males in the best condition prior to emigration embarked on the longest dispersals, and the observed greater survival rate of longer dispersers reflected fitness‐related factors predating transfer. We recommend future dispersal studies collect data sufficient for testing hypotheses associated with condition‐dependent dispersal. Further, we recommend comparison with other large‐mammal systems in which predators persist; perhaps low dispersal mortality for populations of white‐tailed deer in the eastern United States is an artifact of extirpated populations of predators, such as wolves (*Canis lupus*) and cougars (*Puma concolor*).

Dispersal patterns of European roe deer have been well‐studied and offer an interesting contrast. Unlike white‐tailed deer, dispersal rates and distances do not vary by sex (Debeffe et al., 2012). Like white‐tailed deer, dispersal distance distributions are right‐skewed but vary by habitat (Debeffe et al., 2012; Diefenbach et al., 2008), and dispersal appears to be condition‐dependent, such that larger and less parasitized roe deer are more likely to disperse (Debeffe et al., 2012, 2014; Wahlström, 1994). Therefore, in both species those individuals most likely to bear successfully the energetic costs of dispersal are those that disperse. But, the equal sex ratios of dispersal and similar dispersal distance distributions suggest that ultimate causes and consequences of roe deer dispersal may differ from white‐tailed deer.

Animal movement incurs cost, and these costs are typically assumed to increase mortality risk, especially for natal dispersal (Bonte et al., 2012; Ronce, 2007). Although there are certainly costs associated with all phases of dispersal, this study suggests that within some populations and systems, animals can disperse with relatively low direct risk to survival. However, few studies have related natal dispersal to direct mortality risks, especially for large mammals, and it remains to be seen whether low‐risk dispersal is widespread or rare.

## CONFLICT OF INTEREST

None declared.

## AUTHOR CONTRIBUTION

**Eric Shaffer Long:** Conceptualization (equal); Data curation (equal); Formal analysis (supporting); Investigation (equal); Methodology (equal); Writing‐original draft (lead). **Duane R. Diefenbach:** Conceptualization (equal); Data curation (equal); Formal analysis (lead); Funding acquisition (lead); Investigation (equal); Methodology (equal); Project administration (lead); Writing‐review & editing (equal). **Clayton L Lutz:** Data curation (equal); Investigation (equal); Methodology (equal); Writing‐review & editing (equal). **Bret D. Wallingford:** Data curation (equal); Investigation (equal); Methodology (equal); Project administration (equal); Writing‐review & editing (equal). **Christopher S. Rosenberry:** Conceptualization (supporting); Methodology (equal); Project administration (equal); Writing‐review & editing (equal).

## Data Availability

The original data input files are available from the Dryad Digital Repository: doi: 10.5061/dryad.vt4b8gtr8.
